# Influence of Isolated Resistance Exercise on Cardiac Remodeling, Myocardial Oxidative Stress, and Metabolism in Infarcted Rats

**DOI:** 10.3390/antiox12040896

**Published:** 2023-04-07

**Authors:** Eder Anderson Rodrigues, Aline Regina Ruiz Lima, Mariana Janini Gomes, Lidiane Moreira Souza, Thierres Hernani Dias Pontes, Luana Urbano Pagan, Gilson Masahiro Murata, Felipe Cesar Damatto, Igor Carvalho Depra, Amanda Bergamo Gonçalves Castro Rego, David Rafael Abreu Reyes, Leonardo Antonio Mamede Zornoff, Katashi Okoshi, Marina Politi Okoshi

**Affiliations:** 1 Department of Internal Medicine, Botucatu Medical School, Sao Paulo State University (UNESP), Botucatu 18618-687, SP, Brazil; 2 Department of Kinesiology and Sport Management, Texas A&M University, College Station, TX 77845, USA; 3 LIM29, Division of Nephrology, University of Sao Paulo Medical School, Sao Paulo 01246-903, SP, Brazil

**Keywords:** myocardial infarction, physical exercise, rat, ventricular function, metabolism enzymes, NADPH oxidase

## Abstract

Introduction: Exercise is an important therapeutic strategy for preventing and treating myocardial infarction (MI)-induced cardiac remodeling and heart failure. However, the myocardial effects of resistance exercise on infarcted hearts are not completely established. In this study, we investigated the effects of resistance exercise on structural, functional, and molecular cardiac alterations in infarcted rats. Methods: Three months after MI induction or simulated surgery, Wistar rats were assigned into three groups: Sham (*n* = 14); MI (*n* = 9); and exercised MI (MI-Ex, *n* = 13). Exercised rats performed, 3 times a week for 12 weeks, four climbs on a ladder with progressive loads. Cardiac structure and left ventricle (LV) function were analyzed by echocardiogram. Myocyte diameters were evaluated in hematoxylin- and eosin-stained histological sections as the smallest distance between borders drawn across the nucleus. Myocardial energy metabolism, lipid hydroperoxide, malondialdehyde, protein carbonylation, and antioxidant enzyme activities were evaluated by spectrophotometry. Gene expressions of NADPH oxidase subunits were evaluated by RT-PCR. Statistical analyses were performed using ANOVA and Tukey or Kruskal–Wallis and Dunn’s test. Results: Mortality did not differ between the MI-Ex and MI groups. MI had dilated left atrium and LV, with LV systolic dysfunction. Exercise increased the maximum load-carrying capacity, with no changes in cardiac structure or LV function. Myocyte diameters were lower in MI than in Sham and MI-Ex. Lactate dehydrogenase and creatine kinase activity were lower in MI than in Sham. Citrate synthase and catalase activity were lower in MI and MI-Ex than in Sham. Lipid hydroperoxide concentration was lower in MI-Ex than in MI. Nox2 and p22phox gene expressions were higher in MI-Ex than in Sham. Gene expression of Nox4 was higher in MI and MI-Ex than in Sham, and p47phox was lower in MI than in Sham. Conclusion: Late resistance exercise was safe in infarcted rats. Resistance exercise improved maximum load-carrying capacity, reduced myocardial oxidative stress, and preserved myocardial metabolism, with no changes in cardiac structure or left ventricle function in infarcted rats.

## 1. Introduction

Myocardial infarction (MI) is a major contributor to cardiac remodeling and heart failure development and is an important cause of morbidity and mortality worldwide [1]. Cardiac remodeling is defined as gene, molecular, cellular, and interstitial changes in response to heart injury that translate into alterations in cardiac size, geometry, and function [2].

Cardiac remodeling is associated with several metabolic and biochemical changes that may impair energy generation and myocardial function [3]. Oxidative stress, characterized by an imbalance between reactive oxygen species (ROS) and antioxidant defense, is a considerable biochemical alteration commonly observed during cardiac remodeling at both systemic and cardiac levels [4,5]. Nicotinamide adenine dinucleotide phosphate (NADPH) oxidase complex contributes to ROS generation and regulation of metabolic processes and is often activated after MI [6,7].

Physical exercise is an important non-pharmacological strategy for attenuating cardiac remodeling and clinical symptoms in heart failure patients [8]. Aerobic exercise reduces hospitalization and improves cardiovascular function, functional capacity, and quality of life [8,9,10]. In experimental studies, several benefits have been observed, including improvements in myocardial metabolism and antioxidant status [5,11,12]. Despite extensive research on aerobic exercise, the effects of resistance exercise are not well characterized after MI or during heart failure, due to the concern that it was not safe in situations of left ventricular (LV) dilatation [11,13,14,15,16,17]. Only more recently has resistance training been recommended in combination with aerobic exercise as an adjuvant treatment for cardiovascular disease [10,18,19]. The aim of this study was to analyze the effects of isolated and long-term resistance exercise on cardiac remodeling, myocardial metabolism, and oxidative stress in post-infarction rats. Results for echocardiographic data were previously published in a study comparing the effects of aerobic and resistance exercises on skeletal muscle [20].

## 2. Materials and Methods

### 2.1. Experimental Design

Male Wistar rats (200–250 g) were housed in a room under controlled temperature and light–dark cycles. Food and water were supplied *ad libitum*. All experiments were approved by the Ethics Committee of Botucatu Medical School, Sao Paulo State University, UNESP (protocol number 1101).

Myocardial infarction (MI) was induced by ligation of the left anterior descending coronary artery as previously described [21,22,23]. Three months later, rats were submitted for transthoracic echocardiograms, exercise testing, and maximal load-carrying capacity testing and assigned into three groups: Sham (placebo surgery, *n* = 14), MI (*n* = 26), and exercised MI (MI-Ex, *n* = 21). At the end of the experiment, the rats were subjected to the same procedures and euthanized the following day. All rats surviving to the end of the experiment were included in the anatomical and echocardiographic evaluation. Six to ten myocardial samples were randomly chosen for molecular and biochemical assessments.

### 2.2. Maximum Load-Carrying Capacity for Resistance Exercise Training

Exercise was performed on a ladder constructed for rats. Two days after protocol adaptation, each animal was analyzed to determine their maximum load capacity. The test consisted of ladder ascents, with progressive increases in load [24]. Firstly, 75% of rat body weight (BW) was attached to its tail and 15% BW loads were added until the rat could not carry the load to the top of the ladder. The heaviest load that the rat could carry up the full height of the ladder was considered its maximum load capacity. The test was repeated after 45 days to adjust exercise intensity and at the end of the experimental period.

### 2.3. Exercise Protocol

Rats trained 3 days a week for 3 months according to a protocol previously described [20]. There was an initial adaptation period in the first week, when rats were stimulated to climb the ladder with no load. Upon reaching the chamber at the top of the ladder, animals were given a two-minute recovery period and then encouraged to climb the ladder again. This procedure was repeated until the rat climbed the ladder three times without stimulation. The sessions of training consisted of four ladder climbs at 50%, 75%, 90%, and 100% maximum load-carrying capacity with a two-minute recovery period between ascents.

### 2.4. Echocardiographic Evaluation

An echocardiogram was performed before and after the exercise protocol using commercial equipment (Vivid S6, General Electric Medical Systems, Tirat Carmel, Israel) with a 5 to 11.5 MHz multifrequency probe, as a previously described method [25,26,27,28].

### 2.5. Tissue Collection

One day after final echocardiogram, rats were weighed, anesthetized with intraperitoneal sodium pentobarbital (50 mg/kg), and euthanized. After blood collection, the hearts were removed by thoracotomy. Atria and ventricles were dissected and weighed separately. The LV was frozen in liquid nitrogen and stored at −80 °C. The following heart failure decompensation features were evaluated: left atrial thrombi, pleuro-pericardial effusion, hepatic congestion, ascites, right ventricular hypertrophy (right ventricle weight-to-BW ratio higher than 0.8 mg/g), and lung weight [29].

### 2.6. Histological Analysis

LV transverse 8 μm thick sections were cut in a cryostat at −20 °C and stained with hematoxylin and eosin. At least 50 cardiomyocyte diameters were quantified from each rat as the smallest distance between borders drawn across the nucleus [5]. Measurements were performed using a microscope (Leica DM LS; Nussloch, Germany) attached to a computerized imaging analysis system (Media Cybernetics, Silver Spring, MD, USA). Infarction size was measured in LV slices taken 5 to 6 mm from the apex stained with picrosirius red, as previously describe [30].

### 2.7. Metabolic Enzymes Activity

LV samples (~30 mg) were homogenized in 50 mM Tris-HCl, 1 mM EDTA, and protease inhibitor (Sigma Ref. S8820-2TAB), pH 7.4, and centrifuged at 12,000 rpm for 10 min at 4 °C. The supernatant was assayed for maximum activity of the key enzymes that participate in energy metabolism: phosphofructokinase (PFK, EC.2.7.1.11), pyruvate kinase (PK, EC 2.7.1.40), lactate dehydrogenase (LDH, EC 1.1.1.27), citrate synthase (CS, E.C. 4.1.3.7), creatine kinase (CK, EC.2.7.3.2), and carnitine palmitoyltransferase 1 (CPT1, EC 2.3.1.21) [24].

### 2.8. Antioxidant Enzymes Activity

LV samples (~200 mg) were homogenized in 5 mL of cold 0.1 mol/L phosphate buffer, pH 7.0, and centrifuged at 10,000 g for 15 min at 4 °C. The supernatant was assayed for total protein, lipid hydroperoxide, glutathione peroxidase (GSH-Px, E.C.1.11.1.9), catalase (E.C.1.11.1.6.), and superoxide dismutase (SOD, E.C.1.15.1.1.) activities by spectrophotometry [31,32].

### 2.9. Real-Time Quantitative Reverse Transcription Polymerase Chain Reaction (RT-PCR)

Gene expressions of NADPH oxidase subunits Nox2, Nox4, p22phox, and p47phox were analyzed by RT-PCR as previously described [33]. Aliquots of cDNA were then submitted to real-time PCR using a customized assay containing sense and antisense primers and Taqman (Applied Biosystems) probes specific to each gene: Nox2 (Rn00576710m1), Nox4 (Rn00585380m1), p22phox (Rn005773 57m1), and p47phox (Rn00586945m1). Data were normalized using reference genes cyclophilin (Rn00690933m1) and GAPDH (Rn01775763g1). Reactions were performed in triplicate, and expression results were calculated using the CT comparative method (2^−ΔΔCT^).

### 2.10. Statistical Analysis

Results were presented as means and standard deviations or medians and quartiles, according to distribution. Variables were compared by analysis of variance (ANOVA), complemented by Tukey’s test or Kruskal–Wallis and Dunn’s tests. Infarct size was compared using the unpaired Student’s *t* test. The frequency of heart failure features was compared using the Goodman test. Statistical significance was accepted at *p* < 0.05.

## 3. Results

### 3.1. Experimental Groups and Anatomical Variables

After histological analysis, rats with an infarction size < 30% of the total LV area were excluded from the study, including 13 in the MI group and 7 in the MI-Ex group. Five rats died during the protocol, four in MI and one in MI-Ex. Table 1 shows the frequency of heart failure features observed at euthanasia, which did not differ between MI-Ex and MI groups.

**Figure 1:**
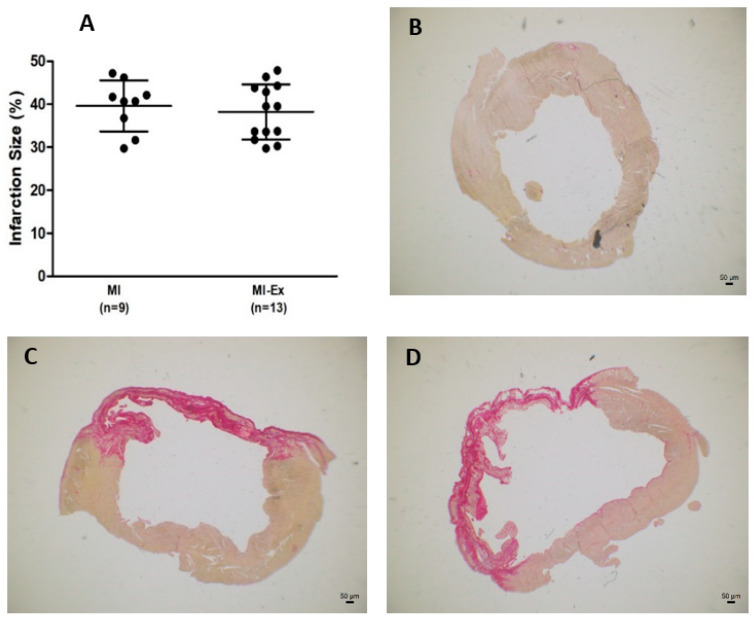
Percentage of left ventricle infarcted area in relation to the total left ventricle area (**A**). Representative histological slides stained with picrosirius red showing left ventricle myocardium from Sham (**B**), myocardial infarction (MI; (**C**)), and exercised myocardial infarction (MI-Ex; (**D**)) groups. Data are means, standard deviations, and individual values; Student’s *t* test; *p* > 0.05.

Anatomical variables are shown in Table 2. Body weight did not differ between groups. RV and atria weights, in absolute and normalized to body weight values, were higher in MI and in MI-Ex than in Sham. Lung weight and lung weight-to-body weight ratio were higher in MI than in Sham. Lung weight in MI-Ex was between that in Sham and MI and did not differ significantly from either group. Infarct size, evaluated by LV histological analysis, did not differ between groups (Figure 1).

### 3.2. Maximum Load-Carrying Capacity

The maximum load-carrying capacity was evaluated before and after the exercise protocol (Table 3). The MI-Ex group had a higher maximum load-carrying capacity than the Sham and MI groups.

### 3.3. Morphometric Analysis

**Figure 2:**
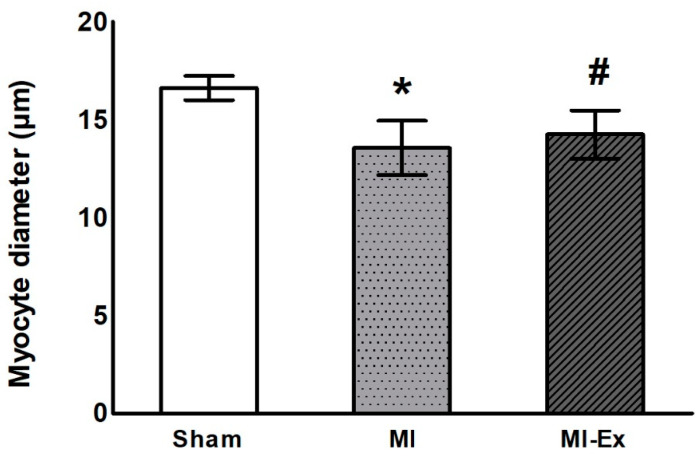
Shortest myocardial fiber diameters. Sham (*n* = 10); MI: myocardial infarction (*n* = 7); MI-Ex: exercised MI (*n* = 10). Data are means ± SD; ANOVA and Tukey; * *p* < 0.05 vs. Sham; # *p* < 0.05 vs. MI.

Figure 2 shows the shortest cardiac fiber diameters; the MI group presented lower values than the Sham and MI-Ex groups.

### 3.4. Echocardiographic Evaluation

Before training, MI and MI-Ex had left atrium dilation, LV hypertrophy, dilation, and systolic dysfunction, with no differences between groups (Table 4 and Table 5).

Final echocardiographic data are presented in Table 6 and Table 7. The same pattern of cardiac chamber hypertrophy and dilation with LV systolic dysfunction was observed in infarcted groups, with no differences between MI-Ex and MI.

### 3.5. Metabolic Enzyme Activity

Table 8 shows energy metabolism enzyme activity. Lactate dehydrogenase (LDH) and creatine kinase (CK) activities were lower in MI than in Sham. Citrate synthase (CS) activity was lower in both infarcted groups than in the Sham group.

### 3.6. Oxidative Stress Evaluation

Antioxidant enzyme activity is shown in Table 9. Catalase activity was lower in the MI and MI-Ex groups than in the Sham group. Glutathione peroxidase activity and superoxide dismutase activity did not differ between groups.

Oxidative stress quantifications are shown in Table 10. Myocardial lipid hydroperoxide concentration was lower in the MI-Ex group than in the MI group. Malonaldehyde concentration and protein carbonylation did not differ between groups.

### 3.7. Real-Time Quantitative Reverse Transcription Polymerase Chain Reaction (RT-PCR)

Gene expressions of NADPH oxidases are shown in Table 11. Nox2 and p22phox subunit expressions were higher in MI-Ex than in Sham. Nox4 subunit expression was higher in MI and MI-Ex than in Sham, and p47phox subunit expression was lower in MI than in Sham.

## 4. Discussion

In this study, we evaluated the effects of physical resistance training on cardiac structural and functional parameters, oxidative stress, antioxidant capacity, and energy metabolism enzyme activity in infarcted rats.

The experimental myocardial infarction model in rodents is often employed, due to the slow development of cardiac remodeling and heart failure and the low cost and high reproducibility of results. However, ventricular dysfunction and cardiac failure only develop in rats with moderate-to-large infarction sizes [4,34]. We previously showed that the minimum infarct sizes able to induce clinical, functional, and structural changes are 40%, 38%, and 36% of the total LV area, respectively [35]. Therefore, we excluded rats with small infarctions in our work, as they are usually excluded from studies evaluating the pathophysiology and treatment of myocardial infarction [36].

Our exercise protocol was effective at increasing muscle strength. Maximum load-carrying capacity was higher in the MI-Ex group than in the Sham and MI groups. Improvement in muscle strength is an important outcome; studies have shown that muscle weakness is associated with higher mortality in heart failure patients [37,38]. In addition to increasing muscle endurance, resistance exercise has also been associated with several beneficial effects, such as an increase in maximal oxygen consumption, functional capacity, and muscle endurance; improvements in quality of life and arterial hypertension control; and prevention of osteoporosis [16,18,39,40,41].

In this study, the MI and MI-Ex groups had an infarction size of approximately 40% of the total LV area. As the infarcted area in rats is established within 24 h post-coronary ligation [42], it is not affected by late treatment. Therefore, a similar infarction area in the MI and MI-Ex groups was important in assuring that both groups had the same degree of cardiac injury before training. Due to the substantial infarction size, MI and MI-Ex groups presented left atrium dilation LV hypertrophy, dilation, and systolic dysfunction before the exercise protocol. Despite the large infarcted area, exercise was safely tolerated, and the MI-Ex group had a low mortality rate. Understanding the benefits of physical exercise post-myocardial infarction has mostly resulted from studies with aerobic exercise [43] because physicians and researchers were worried that the increased intravascular pressure, which occurs during weightlifting, could augment LV systolic pressure and further dilatate LV [16,17]. In fact, the intense physiologic demand that occurs in athletes may induce cardiac dilation and hypertrophy, which may be similarly observed in different forms of pathologic cardiac remodeling [44,45]. More recently, experimental studies have shown that resistance exercise is safe for infarcted rodents [11,13,14,15].

Resistance exercise did not change cardiac remodeling. The final echocardiogram showed no differences between MI-Ex and MI, and both groups still had the same pattern of change in the left chambers as seen before exercise. The fact that the shortest myocyte diameter was lower in MI than in Sham and MI-Ex reinforced the fact that resistance exercise was not associated with additional LV dilation. A few studies have observed slight improvements in cardiac remodeling following resistance exercise in infarcted rats [11,13,14,15]. In these studies, exercise was initiated within one to five weeks after inducing infarction. In our study, we were concerned that moderate resistance exercise practiced early after infarction, when the LV fibrotic scar has not yet consolidated [42], could further dilatate LV. Therefore, exercise was probably initiated late, when the LV injury was well established, thus preventing a reverse remodeling process. Additional studies are needed to clarify the best time to initiate exercise after myocardial infarction.

The myocardium can use different energy substrates [46]. Complex enzymatic mechanisms regulate metabolic processes generating energy [6]. The main myocardial sources of adenosine triphosphate (ATP) are oxidative phosphorylation and glycolysis, which contribute 95% and 5%, respectively, to ATP generation [47]. Under physiological conditions, fatty acids are the main energy source, contributing to approximately 60% of mitochondrial ATP synthesis and glucose, and lactate oxidation contributes to almost 40% of synthesized energy; ketone bodies and amino acids have a small contribution [47,48]. During cardiac remodeling, metabolic flexibility is impaired, and the fuel preference switches to glucose [3,49]. Here, we observed that infarction changed the activity of key enzymes in energy metabolism. A reduction in creatine kinase activity was observed in experimental heart failure [50]. Therefore, it was interesting to observe that resistance exercise preserved creatine kinase and lactate dehydrogenase activities. As the role of chronic resistance exercise on myocardial metabolism was not previously evaluated, additional studies with a large panel of energy biomarkers could help to clarify this issue.

Oxidative stress is characterized by an imbalance between ROS production and antioxidant capacity [51]. At physiological concentrations, ROS are important in preserving cell signaling and survival, which are jeopardized under high ROS levels [51]. Cardiac remodeling and heart failure are associated with increased systemic and cardiac oxidative stress [4,32]. We evaluated myocardial oxidative stress markers—lipid hydroperoxide, malondialdehyde, and protein carbonylation. The fact that the lipid hydroperoxide concentration was lower in MI-Ex than in MI showed that exercise reduced oxidative stress and suggested the importance of assessing several oxidative stress markers. Concerning antioxidant enzyme activity, catalase activity was lower in both MI and MI-Ex than in Sham. Therefore, exercise reduced oxidative stress with no influence on antioxidant enzyme activity. In skeletal muscles, regular resistance training increases the abundance/activity of antioxidant enzymes, therefore increasing resistance to oxidative stress [52]. Our data suggest that the effects of resistance exercise on myocardium could differ from those on skeletal muscles.

The NADPH oxidase complex contributes to ROS generation and cardiac metabolic process regulation [6]. The Nox2 and Nox4 subunits are usually up-regulated during cardiac remodeling and play different roles in the heart [6]. Nox2 activation depends on the binding of regulatory units, including p47phox, to the Nox2–p22phox complex and is associated with deleterious cardiac signaling. On the other hand, Nox4 is constitutively active and drives protective signaling pathways [6]. In this study, the Nox4 gene expression was higher in both MI and MI-Ex groups than in Sham, showing that it was not modulated by exercise. Unexpectedly, Nox2 and p22phox were higher in MI-Ex than in Sham. No differences between MI-Ex and MI were found. Exercise has a dual effect on systemic and skeletal muscle oxidative stress. Acute and intense exercise is associated with increased ROS formation, while regular exercise may cause a slight increase in ROS production, which induces beneficial cell adaptations [43,53]. Since our rats practiced chronic regular exercise, we expected them to present increased antioxidant enzyme activity and reduced Nox2 subunit expression, which were not observed. We did not evaluate NADPH subunit activities in this study. As the Nox2–p22phox complex activation required p47phox phosphorylation, and as p47phox gene expression did not change, we could not assume that Nox2 activity increased in our rats. A recent study showed that genetic ablation of p47phox inhibited Nox2-induced superoxide production in the hearts of mice treated with angiotensin-II [54]. Therefore, additional studies are needed to evaluate whether NADPH oxidase subunits have the same behavior as gene expression.

Resistance exercise was safe, reduced myocardial stress, and preserved myocardial metabolism in infarcted rats. However, the biochemical and metabolic improvements were not translated into better cardiac remodeling or ventricular function. It is possible that the timing of the evaluation was not appropriate for showing the influence of reduced oxidative stress and improving metabolism on cardiac remodeling. A limitation of our study is that, due to the well-known concern that hormonal changes in females may influence the results, only male rats were included in this experiment.

## 5. Conclusions

Resistance exercise is safe, reduces myocardial stress, and preserves myocardial metabolism independently of cardiac remodeling changes in infarcted rats.

## Figures and Tables

**Table 1:** Heart failure features frequency in myocardial infarction groups.

	Frequency (*n*, %)	
	MI (*n* = 9)	MI-Ex (*n* = 13)
Atria thrombi	11.1 (1)	23.1 (3)
Pleuro-pericardial effusion	11.1 (1)	30.8 (4)
Hepatic congestion	33.3 (3)	23.1 (3)
Right ventricular hypertrophy	33.3 (3)	30.8 (4)
Ascites	22.2 (2)	15.4 (2)

MI: myocardial infarction; MI-Ex: exercised MI; *n*: number of animals. Goodman test; *p* > 0.05.

**Table 2:** Anatomical parameters.

	Sham (*n* = 14)	MI (*n* = 9)	MI-Ex (*n* = 13)
BW (g)	552 (527–582)	531 (511–572)	526 (476–570)
RV (g)	0.24 (0.21–0.26)	0.36 (0.30–0.43) *	0.34 (0.30–0.50) *
RV/BW (mg/g)	0.43 (0.41–0.45)	0.71 (0.52–0.86) *	0.65 (0.56–0.94) *
Atria (mg)	0.10 (0.09–0.14)	0.15 (0.11–0.31) *	0.16 (0.14–0.19) *
Atria/BW (mg/g)	0.19 (0.16–0.32)	0.32 (0.22–0.54) *	0.33 (0.24–0.49) *
Lung (g)	1.98 ± 0.68	2.98 ± 0.89 *	2.64 ± 0.70
Lung/BW (mg/g)	3.65 (3.19–3.70)	5.18 (4.43–7.24) *	4.82 (3.77–5.27)

MI: myocardial infarction; MI-Ex: exercised MI; *n*: number of animals; BW: body weight; RV: right ventricle weight. Data are means ± SD or medians and percentiles; ANOVA and Tukey or Kruskal–Wallis and Dunn; * *p* < 0.05 vs. Sham.

**Table 3:** Maximum load-carrying capacity.

	Sham (*n* = 4)	MI (*n* = 7)	MI-Ex (*n* = 13)
Initial (g)	416 ± 90	281 ± 70 *	342 ± 81
Final (g)	306 ± 144	349 ± 143	617 ± 169 *^,#,&^

MI: myocardial infarction; MI-Ex: exercised MI; Initial: before exercise training; Final: at the end of the exercise protocol. Data are means ± SD; ANOVA and Tukey; * *p* < 0.05 vs. Sham; ^#^ *p* < 0.05 vs. MI; ^&^ *p* < 0.05 vs. Initial.

**Table 4:** Echocardiographic structural data before the exercise protocol.

	Sham (*n* = 14)	MI (*n* = 9)	MI-Ex (*n* = 13)
BW (g)	471 ± 47	458 ± 43	447 ± 47
LVDD (mm)	8.07 ± 0.44	10.53 ± 0.69 *	10.16 ± 1.07 *
LVDD/BW (mm/kg)	17.3 ± 1.39	23.2 ± 2.68 *	22.8 ± 2.56 *
LVSD (mm)	4.10 (3.81–4.30)	8.16 (7.99–8.62) *	7.89 (7.23–8.55) *
DPWT (mm)	1.33 (1.27–1.37)	1.65 (1.54–1.95) *	1.65 (1.49–1.74) *
AO (mm)	4.01 (3.83–4.01)	3.94 (3.78–4.01)	3.83 (3.61–4.01)
LA (mm)	5.29 (5.11–5.66)	7.66 (6.70–8.27) *	6.75 (6.27–7.41) *
LA/AO	1.35 (1.32–1.39)	1.94 (1.67–2.27) *	1.80 (1.55–1.99) *
LA/BW (mm/kg)	11.6 (10.4–12.3)	17.4 (13.9–19.5) *	15.3 (13.3–17.5) *
End-diast. area (mm^2^)	44.2 ± 7.43	89.5 ± 14.7 *	80.5 ± 14.3 *
End-sist. area (mm^2^)	15.6 (12.5–18.0)	61.6 (55.6–72.1) *	52.2 (43.1–58.9) *

MI: myocardial infarction; MI-Ex: exercised MI; *n*: number of animals; LVDD and LVSD: left ventricular (LV) diastolic and systolic diameters, respectively; BW: body weight; DPWT: LV diastolic posterior wall thickness; AO: aorta diameter; LA: left atrial diameter; End-diast. area and End-sist. area: LV end-diastolic and end-systolic areas, respectively. Data are means ± SD or medians and percentiles; ANOVA and Tukey or Kruskal–Wallis and Dunn; * *p* < 0.05 vs. Sham.

**Table 5:** Echocardiographic left ventricular functional data before the exercise protocol.

	Sham (*n* = 14)	MI (*n* = 9)	MI-Ex (*n* = 13)
HR (bpm)	292 ± 25	295 ± 26	275 ± 24
EF	0.86 (0.84–0.89)	0.51 (0.43–0.57) *	0.50 (0.42–0.58) *
PWSV (mm/s)	40.5 ± 5.58	24.5 ± 7.67*	28.6 ± 8.37 *
Tei index	0.47 (0.44–0.53)	0.74 (0.63–0.89) *	0.76 (0.63–0.81) *
FAC (%)	65.7 ± 5.52	28.6 ± 8.19 *	33.7 ± 7.59 *
Mitral E (cm/s)	80.3 ± 7.53	99.1 ± 16.5 *	88.5 ± 18.3
Mitral A (cm/s)	47 (45–55)	44 (21–64)	42 (23–58)
E/A	1.65 (1.47–1.82)	2.02 (1.44–5.27)	1.63 (1.41–4.70)
IVRT (ms)	26 (22–26)	30 (26–33)	30 (25–32)
TDI S’ (average, cm/s)	3.47 ± 0.33	3.13 ± 0.36	2.90 ± 0.46 *
TDI E’ (average, cm/s)	4.15 ± 0.72	4.10 ± 0.52	3.76 ± 0.50
E/TDI E’ (average)	19.8 ± 3.69	24.5 ± 5.38 *	24.5 ± 6.44 *

MI: myocardial infarction; MI-Ex: exercised MI; *n*: number of animals; HR: heart rate; EF: ejection fraction; PWSV: posterior wall shortening velocity; Tei index: myocardial performance index; FAC: fractional area change; E/A: ratio between early (E)-to-late (A) diastolic mitral inflow; IVRT: isovolumetric relaxation time; TDI S’: tissue Doppler imaging (TDI) of the systolic velocity of the mitral annulus; TDI E’: TDI of the early diastolic velocity of mitral annulus. Data are means ± SD or medians and percentiles; ANOVA and Tukey or Kruskal–Wallis and Dunn; * *p* < 0.05 vs. Sham.

**Table 6:** Echocardiographic structural data at the end of the experiment.

	Sham (*n* = 14)	MI (*n* = 9)	MI-Ex (*n* = 13)
LVDD (mm)	8.34 ± 0.43	11.1 ± 0.88 *	10.9 ± 0.82 *
LVDD/BW (mm/kg)	15.1 (14.3–15.9)	21.5 (18.5–22.2) *	20.8 (19.8–22.3) *
LVSD (mm)	4.09 ± 0.38	8.96 ± 1.33 *	8.62 ± 1.16 *
DPWT (mm)	1.42 (1.38–1.43)	1.80 (1.64–2.01) *	1.69 (1.60–1.73) *
AO (mm)	4.21 ± 0.15	4.01 ± 0.23	4.06 ± 0.27
LA (mm)	5.68 ± 0.46	8.23 ± 1.30 *	7.80 ± 1.26 *
LA/AO	1.38 (1.24–1.44)	2.22 (1.63–2.34) *	1.86 (1.78–2.23) *
LA/BW (mm/kg)	10.1 (9.21–11.1)	15.9 (12.0–17.5) *	14.7 (12.8–17.1) *
End-diast. area (mm^2^)	49 (47–51)	90 (85–96) *	94 (80–106) *
End-sist. area (mm^2^)	15 (14–18)	65 (56–72) *	60 (47–76) *

MI: myocardial infarction; MI-Ex: exercised MI; *n*: number of animals; LVDD and LVSD: left ventricular (LV) diastolic and systolic diameters, respectively; BW: body weight; DPWT: LV diastolic posterior wall thickness; AO: aorta diameter; LA: left atrial diameter; End-diast. area and End-sist. area: LV end-diastolic and end-systolic areas, respectively. Data are means ± SD or medians and percentiles; ANOVA and Tukey or Kruskal–Wallis and Dunn; * *p* < 0.05 vs. Sham.

**Table 7:** Echocardiographic left ventricular functional data at the end of the experiment.

	Sham (*n* = 14)	MI (*n* = 9)	MI-Ex (*n* = 13)
HR (bpm)	280 ± 39	300 ± 32	292 ± 21
EF	0.88 (0.87–0.90)	0.46 (0.40–0.54) *	0.53 (0.42–0.59) *
PWSV (mm/s)	42 ± 6.2	25 ± 9.4 *	28 ± 6.2 *
Tei index	0.47 ± 0.06	0.62 ± 0.14 *	0.66 ± 0.12 *
FAC (%)	67.3 ± 4.90	29.6 ± 8.92 *	34.4 ± 12.2 *
Mitral E (cm/s)	77 (74–86)	102 (79–120)	78 (72–121)
Mitral A (cm/s)	51 ± 15	39 ± 28	47 ± 21
E/A	1.71 (1.41–1.80)	4.27 (1.30–5.99)	1.41 (1.24–5.53)
IVRT (ms)	25 ± 3.7	27 ± 5.2	27 ± 4.3
TDI S’ (average, cm/s)	3.55 ± 0.36	2.80 ± 0.49 *	2.93 ± 0.45 *
TDI E’ (average, cm/s)	4.16 ± 0.68	4.18 ± 0.76	3.92 ± 0.56
E/TDI E’ (average)	18 (16–21)	23 (20–25)	22 (19–28)

MI: myocardial infarction; MI-Ex: exercised MI; *n*: number of animals; HR: heart rate; EF: ejection fraction; PWSV: posterior wall shortening velocity; Tei index: myocardial performance index; E/A: ratio between early (E)-to-late (A) diastolic mitral inflow; IVRT: isovolumetric relaxation time; TDI S’: tissue Doppler imaging (TDI) of the systolic velocity of the mitral annulus; TDI E’: TDI of the early diastolic velocity of the mitral annulus. Data are means ± SD or medians and percentiles; ANOVA and Tukey or Kruskal–Wallis and Dunn; * *p* < 0.05 vs. Sham.

**Table 8:** Myocardial energy metabolism.

	Sham (*n* = 9)	MI (*n* = 9)	MI-Ex (*n* = 10)
PFK (nmol/min·g of protein)	23.7 (20.5–28.3)	22.1 (15.0–27.9)	25.3 (22.9–28.3)
PK (nmol/min·g of protein)	235 ± 38.8	204 ± 23.1	225 ± 40.6
LDH (nmol/min·g of protein)	1241 ± 131	1034 ± 112 *	1129 ± 157
CS (umol/min·g of protein)	293 ± 33	207 ± 48 *	232 ± 55 *
CK (nmol/min·g of protein)	30.8 ± 4.93	24.8 ± 4.91 *	28.8 ± 3.86
CPT1 (nmol/min·g of protein)	16.8 ± 4.46	22.2 ± 6.56	22.6 ± 6.76

MI: myocardial infarction; MI-Ex: exercised MI; *n*: number of animals; PFK: phosphofructokinase; PK: pyruvato kinase; LDH: lactate dehydrogenase; CS: citrate synthase; CK: creatine kinase; CPT1: carnitine palmitoyltransferase 1. Data are mean ± SD or medians and percentiles; ANOVA and Tukey or Kruskal–Wallis and Dunn; * *p* < 0.05 vs. Sham.

**Table 9:** Myocardial antioxidant enzyme activity.

	Sham (*n* = 7)	MI (*n* = 8)	MI-Ex (*n* = 8)
Superoxide dismutase (nmol/g of tissue)	7.49 (6.22–8.87)	6.67 (5.67–7.25)	5.78 (5.36–6.13)
Catalase (µmol/g of tissue)	54.2 ± 8.31	47.3 ± 10.34 *	40.8 ± 6.97 *
Glutathione peroxidase (nmol/g of protein)	30.0 ± 5.70	29.3 ± 6.95	29.7 ± 7.66

MI: myocardial infarction; MI-Ex: exercised MI; *n*: number of animals. Data are mean ± SD or medians and percentiles; ANOVA and Tukey or Kruskal–Wallis and Dunn; * *p* < 0.05 vs. Sham.

**Table 10:** Myocardial oxidative stress.

	Sham (*n* = 7)	MI (*n* = 8)	MI-Ex (*n* = 8)
Malondialdehyde (nmol/mg of protein)	4.98 (4.77–5.29)	5.46 (3.71–7.89)	4.88 (4.36–9.82)
Protein carbonylation (nmol/mg of protein)	3.12 (3.05–3.26)	3.02 (2.82–3.14)	3.14 (3.10–3.21)
Lipid hydroperoxide (nmol/g of tissue)	160 (128–187)	204 (182–244)	128 (118–152) #

MI: myocardial infarction; MI-Ex: exercised MI; *n*: number of animals. Data are medians and percentiles; Kruskal–Wallis and Dunn; # *p* < 0.05 versus MI.

**Table 11:** Myocardial gene expression of NADPH oxidases.

	Sham (*n* = 8)	MI (*n* = 6)	MI-Ex (*n* = 8)
Nox2	0.98 (0.87–1.07)	1.43 (1.19–2.30)	1.96 (1.05–2.76) *
Nox4	0.97 ± 0.43	1.60 ± 0.41 *	1.57 ± 0.58 *
p22phox	0.99 (0.96–1.03)	1.06 (0.89–1.20)	1.32 (1.02–1.51) *
p47phox	0.98 (0.78–1.12)	0.66 (0.60–0.68) *	0.81 (0.58–0.95)

MI: myocardial infarction; MI-Ex: exercised MI; *n*: number of animals. Data are mean ± SD or medians and percentiles; ANOVA and Tukey or Kruskal–Wallis and Dunn; * *p* < 0.05 vs. Sham.

## Data Availability

All data generated or analyzed during this study are included in this manuscript.
